# PI-RADSv2.1 combined with PSA density for optimizing prostate biopsy decisions: a retrospective analysis

**DOI:** 10.3389/fonc.2025.1602412

**Published:** 2025-07-04

**Authors:** Yuchun Li, Siran Wang, Jianqiu Wang, Xin Qi, Tianran Liu, Xiaodong He, Yu Zhang, Yi Zhu, Yunfu Zeng

**Affiliations:** ^1^ Department of Radiology, First People’s Hospital of Yibin, Sichuan, China; ^2^ Philips Healthcare (China), Shanghai, China

**Keywords:** biopsy, indications, multiparametric magnetic resonance imaging, prostate cancer, prostate imaging reporting and data system, prostate-specific antigen

## Abstract

**Objective:**

This study aimed to explore the application value of the Prostate Imaging Reporting and Data System version 2.1 (PI-RADS v2.1) combined with prostate-specific antigen density (PSAD) for guiding prostate biopsy, with the goal of improving biopsy positivity rates and reducing unnecessary procedures.

**Methods:**

A retrospective analysis was conducted on data from 462 patients (44-89years) who underwent prostate biopsy. Univariate and multivariate logistic regression analyses were used to identify independent risk factors for clinically significant prostate cancer (csPCa). Independent risk factors were explored to establish a diagnostic model for csPCa and indications for prostate biopsy. The diagnostic performance was evaluated by receiver operating characteristic (ROC) curve analysis, assessing sensitivity, specificity, positive predictive value, negative predictive value, biopsy avoidance rate, and missed diagnosis rate.

**Results:**

Univariate and multivariate logistic regression analyses indicated that PI-RADS v2.1 score [*P* < 0.001; odds ratio (OR) =9.779; 95% confidence interval (CI) = 5.849-16.349] and PSA density (PSAD) [*P* < 0.001; OR = 6.128; 95% CI = 2.292-16.386] were independent risk factors for csPCa. The combined PI-RADS v2.1 and PSAD approach exhibited excellent diagnostic performance (AUC = 0.966), with sensitivity and specificity of 92.4% and 91.6%, respectively. The threshold for diagnosing csPCa was a PI-RADS v2.1 score of ≥ 4 and PSAD ≥ 0.30 ng/(mL·cm³). Specifically, no csPCa was detected among patients with PI-RADS ≤ 2 and PSAD < 0.30 ng/(mL·cm³), indicating these biopsies could be safely avoided. Similarly, for patients with a PI-RADS v2.1 score of 3 and PSAD < 0.15 ng/(mL·cm³), the csPCa detection rate was also zero, supporting biopsy avoidance in these cases.

**Conclusion:**

The use of the PI-RADS v2.1 score combined with PSAD is recommended as an indication for prostate biopsy: (1) For patients with PI-RADS scores of 1-2, biopsy can be avoided if PSAD is < 0.30 ng/(mL·cm³), whereas biopsy is recommended if PSAD is ≥ 0.30 ng/(mL·cm³). (2) For patients with a PI-RADS score of 3, biopsy can be avoided if PSAD is < 0.15 ng/(mL·cm³), but it is recommended if PSAD is ≥ 0.15 ng/(mL·cm³). (3) Patients with a PI-RADS score of 4 are recommended for biopsy in all cases. (4) For patients with a PI-RADS score of 5, biopsy is recommended if PSAD is < 2.00 ng/(mL·cm³), but empirical initiation of treatment without biopsy may be considered if PSAD ≥ 2.00 ng/(mL·cm³), subject to ethics committee approval. Using these criteria, 40% (186/462) of patients in this study could potentially avoid prostate biopsy.

## Introduction

Prostate cancer (PCa) is a prevalent and often fatal malignancy among men (1). It is characterized by an insidious onset, substantial variability between individuals, diagnostic challenges, and a propensity for late-stage metastasis, which establishes it as a remarkable area of clinical research. Although prostate biopsy is regarded as the diagnostic gold standard, it is an invasive procedure with risks of complications such as infections and bleeding (2). Therefore, refining biopsy criteria to improve diagnostic accuracy while minimizing unnecessary procedures is essential.

Traditional guidelines typically rely on prostate-specific antigen (PSA) testing and digital rectal examination to determine the need for biopsy. However, these methods can yield a high false-positive rate, increasing the likelihood of unnecessary biopsies (3). Multiparametric MRI (mpMRI) remarkably bolsters diagnostic precision by providing detailed information on lesion location, tissue characteristics, and blood flow (4). The Prostate Imaging Reporting and Data System version 2.1 (PI-RADS v2.1) offers standardized diagnostic guidelines for mpMRI interpretation, quantifying and categorizing imaging features to aid clinicians in pinpointing potential cancer risk areas (5–9). This scoring system enhances the consistency and accuracy of PCa diagnoses. Compared with PI-RADS v2.0, the PI-RADS v2.1 system enhances the evaluation of T2-weighted imaging (T2WI) for the transition zone (TZ) and diffusion-weighted imaging (DWI) for the peripheral zone (PZ), improving its diagnostic value (10, 11).

Recent studies underscore the clinical benefit of combining PI-RADS v2.1 scoring with PSA derivatives, such as PSA density (PSAD), in determining biopsy necessity (12–16). PSAD is particularly useful in accounting for the effect of prostate volume on PSA levels, thereby more accurately reflecting PSA changes caused by prostate lesions (17). Specific thresholds, such as avoiding biopsies when PI-RADS scores are below 3 and PSAD is below 0.15 ng/(mL·cm³), help reduce unnecessary interventions (15). Additionally, findings indicate a correlation of a PI-RADS score of ≤2, or a score of 3 with PSAD below 0.33 ng/(mL·cm³) with a low likelihood of PCa, suggesting that biopsies in these cases may be unwarranted (16).

Despite these advances, consensus on biopsy decisions based on PI-RADS scores of 3–5 still remains obscure. With more patients undergoing MRI before biopsy, prioritizing PI-RADS scoring as a primary criterion—complemented by PSA levels for further stratification—may help identify those who genuinely need a biopsy.

This study aimed to rigorously assess the integration of PI-RADS v2.1 scoring with PSAD to develop a new, comprehensive set of standardized biopsy guidelines to reduce unnecessary procedures, optimize clinical practice, and enhance both patient safety and diagnostic effectiveness.

## Materials and methods

### Study population

**Figure 1:**
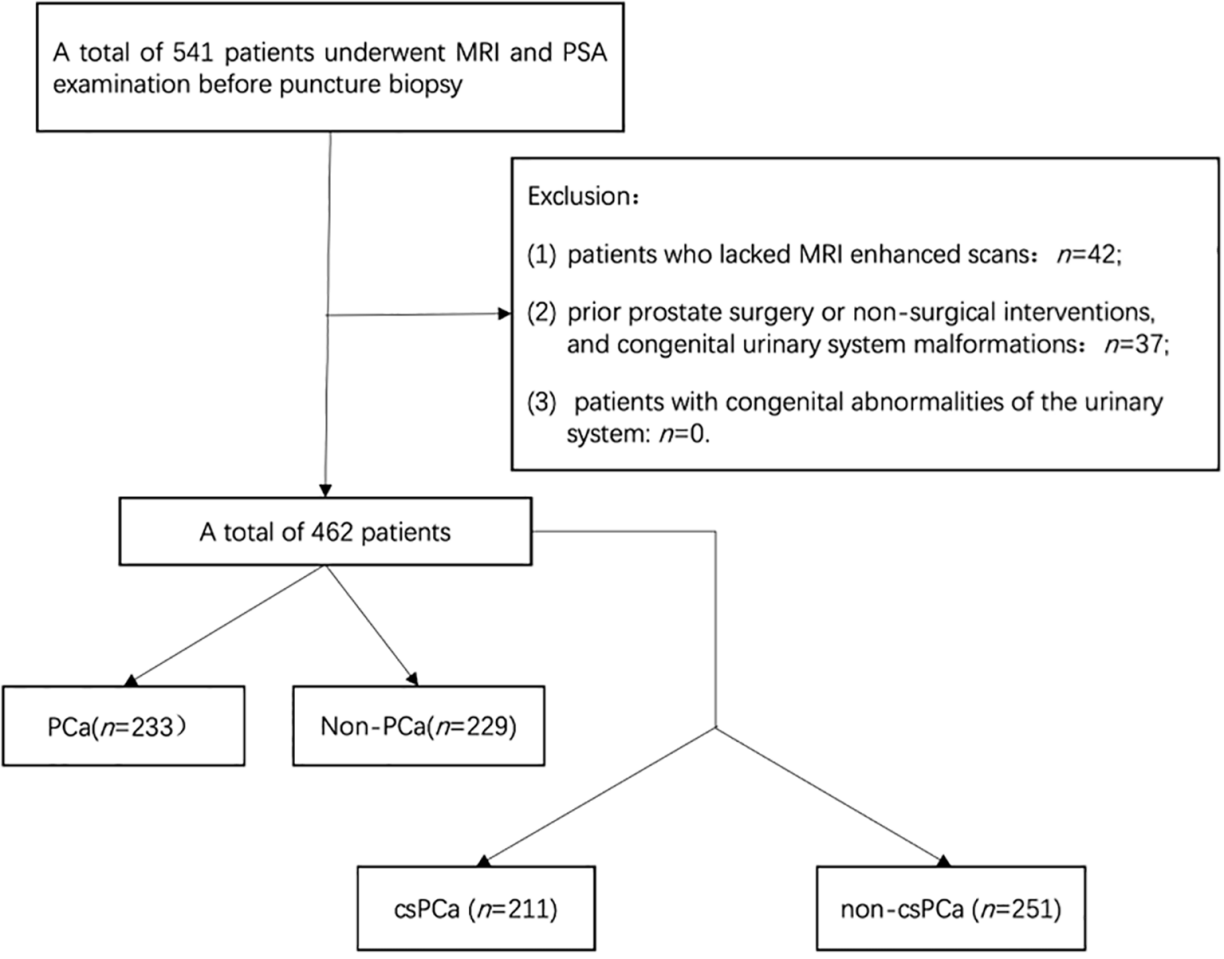
Flowchart of the patient enrollment process.

This retrospective study used clinical data from 462 continuous patients at our hospital from January 2020 to October 2024. Ethical approval was obtained from our local ethics committee, and informed consent was not required. The inclusion criteria were as follows: (1) patients who had undergone both multiparametric magnetic resonance imaging (mpMRI) and PSA-related testing [including total PSA (tPSA), free prostate-specific antigen (fPSA), the ratio of free to total PSA (f/tPSA), and PSAD] prior to biopsy, and (2) patients who had their initial prostate biopsy performed with comprehensive pathologic results available. The exclusion criteria were as follows: (1) incomplete clinical data or MRI examination, (2) patients who had previously undergone prostate surgery or received nonsurgical interventions affecting the prostate, and (3) patients with congenital abnormalities of the urinary system. The process of patient selection based on these criteria is illustrated in **Figure 1**.

### MRI examination protocol

A 3.0 Tesla MRI (Philips, Amsterdam, Netherlands) scanner was used with a 32-channel phased array body coil as the receiving coil. The coil center is located 5 cm above the symphysis pubis. The scan included the prostate and seminal vesicle glands. The scanning sequences included axial T1-weighted imaging (T1WI), axial T2-weighted imaging (T2WI), sagittal T2WI,coronal T2WI, diffusion-weighted imaging (DWI) (b values of 0, 800, 1500 s/mm²), and dynamic contrast-enhanced imaging(DCE). The parameters of each sequence are shown in **Table 1**.

**Table 1:** MRI sequence parameters TR, TE, and FOV for T1WI, DWI, and DCE.

Variable	T1WI	T2WI	DWI	DCE
TR (ms)	527	4884	3932	4
TE (ms)	10	100	69	1.87
Slice thickness(mm)	3	3	3	3
Interslice gap(mm)	0.3	0.3	0.3	-1.5
Matrix size	256×202	420×378	108×113	148×148
FOV (mm)	230×220	230×220	200×210	220×220

DCE, Dynamic contrast-enhanced imaging; DWI, diffusion-weighted imaging; FOV, field of view; T1WI, T1-weighted imaging; T2WI, T2-weighted imaging; TE, echo time; TR, repetition time.

### Image analysis and PI-RADS v2.1 scoring

All cases were diagnosed by 2 radiologists specializing in abdominal diseases with more than 15 years of experience, blinded to the pathologic results. Independent scoring and recording of all MRI images were performed according to the PI-RADS v2.1 scoring criteria. In case of discrepancies, the final scores were determined by consensus between 2 physicians. TZ lesions were primarily evaluated on T2WI sequences, with DWI sequences as secondary, whereas PZ lesions were primarily evaluated on DWI sequences, with contrast-enhanced imaging and T2WI sequences as secondary. The highest score in the TZ or PZ was used as the final PI-RADS v2.1 score for each case.

Prostate volume (PV) = Anteroposterior diameter × Transverse diameter × Superoinferior diameter × 0.52(The anteroposterior and superior-inferior diameters were measured in the sagittal position on T2WI, and the transverse diameter was measured in the axial position on T2WI).

PSAD = tPSA/PV

### Biopsy methods and pathologic results

All patients underwent a transrectal ultrasound-guided 12-core biopsy system. Additional 2–3 cores of MRI-TRUS fusion targeted biopsy were performed for suspicious lesions. All cases were diagnosed by 2 pathologists, In case of discrepancies, the final scores were determined by consensus between 2 physicians. Pathological results with Gleason scores ≥3 + 4 were considered clinically significant prostate cancer (csPCa), and Gleason scores ≤3 + 3 were considered clinically insignificant prostate cancer (InsPCa).

### Statistical analysis

Statistical analysis was performed using SPSS 26.0 software (IBM,America). Normally distributed continuous data were expressed as mean ± standard deviation and compared using t tests. In contrast, non-normally distributed continuous data were expressed as median and quartiles [M (P25-P75)] and compared using Mann–Whitney U tests. Count data were expressed as numbers (percentages) and compared using chi-square tests. Univariate and multivariate logistic regression analyses were conducted to identify risk factors for PCa. The Youden index was used to determine the optimal threshold for biopsy positivity (including csPCa and insPCa) and csPCa. Sensitivity, specificity, negative predictive value (NPV), and positive predictive value (PPV) for individual or combined diagnosis of PCa were calculated based on the optimal threshold. A *P* value <0.05 indicated a statistically significant difference.

## Results

### General characteristics of biopsy groups

Comparison of general information between positive and negative biopsy groups: Among the 462 patients, 233 had PCa and 229 had non-PCa, with a biopsy positivity rate of approximately 50.4%. Among the 462 patients, 211 had csPCa and 251 had non-csPCa. The detection rates of PCa and csPCa in groups with PI-RADS v2.1 scores of 1-2, 3, 4, and 5 were as follows: 3.13%, 9.68%, 55.77%, and 94.70% for PCa and 3.13%, 3.22%, 446.15%, and 92.35% for csPCa, respectively. Statistically significant differences were observed in patient’s age, tPSA, fPSA, PV, PSAD, and PI-RADS v2.1 scores between the positive and negative groups, as well as between the csPCa and non-csPCa groups (**Tables 2**).

**Table 2:** General information on PCa, non-PCa, csPCa and non-csPCa groups.

Variables	PCa	non-PCa	*P* value	csPCa	non-csPCa	*P* value
	*n*= 233	*n*= 229		*n*= 211	*n*= 251	
Age (years)	71.63±7.65	69.20±8.14	0.001^*^	71.46±7.63	69.55±8.18	0.010^*^
tPSA (ng/mL)	41.02 (17.87, 183.28)	11.28 (7.83, 16.20)	<0.001^*^	49.99 (23.22, 195.08)	11.20 (7.48, 16.21)	<0.001^*^
fPSA (ng/mL)	5.47 (2.18, 22.49)	1.63 (1.01, 3.00)	<0.001^*^	6.33 (2.56, 29.29)	1.634 (1.01, 3.05)	<0.001^*^
fPSA/tPSA	0.13(0.10, 0.17)	0.16(0.11, 0.23)	<0.001^*^	0.13 (0.10, 0.17)	0.16 (0.11, 0.23)	<0.001^*^
PV (cm^3^)	38.37 (28.13, 60.65)	59.83 (41.60, 79.0)	<0.001^*^	38.25 (27.64, 57.73)	58.50 (39.88, 78.55)	<0.001^*^
PSAD (ng/(mL·cm³))	1.23 (0.48, 3.45)	0.18 (0.12, 0.31)	<0.001^*^	1.32 (0.59, 4.06)	0.18 (0.12, 0.32)	<0.001^*^
PI-RADSv2.1 score (cases/%)			<0.001^*^			<0.001^*^
1-2	2 (3.13)	62 (96.87)		2 (3.13)	62 (96.87)	
3	12 (9.68)	112 (90.32)		4 (3.22)	120 (96.77)	
4	58 (55.77)	46 (44.23)		48 (46.15)	56 (53.85)	
5	161 (94.70)	9 (5.30)		157 (92.35)	13 (7.65)	

PCa, prostate cancer; csPCa, Clinically significant prostate cancer; PI-RADS v2.1, Prostate Imaging Reporting and Data System version 2.1; fPSA, Free prostate-specific antigen (PSA); tPSA, total PSA; f/tPSA, the ratio of free to total PSA; PSAD, PSA density, PV, prostate volume. *P<0.05.

### Diagnostic accuracy of PI-RADS v2.1 and PSAD for biopsy positivity and csPCa

Univariate logistic regression analysis indicated that patient age, tPSA, fPSA, PV, PSAD, and PI-RADS v2.1 scores were statistically significant risk factors for biopsy positivity [all *P* < 0.05, with odds ratio (OR) of 1.001,1.015, 1.073, 0.997,2.368, and 1.115, respectively].Similarly, patient age, tPSA, fPSA, f/tPSA, PV, PSAD, and PI-RADS v2.1 scores were vital risk factors for csPCa (all *P* < 0.05, with OR of 1.031, 1.058, 1.172, 0.021, 0.989, 21.466, and 13.945, respectively) (**Table 3**).Multivariate logistic regression analysis revealed that PI-RADS v2.1 scores [*p* < 0.001; OR = 7.728; 95% confidence interval (CI) = 4.955-12.055)], PSAD (P = 0.001; OR = 4.478; 95% CI = 1.795-11.170) and PV (*p* = 0.026; OR = 0.986; 95% CI = 0.974-0.998) were independent risk factors for biopsy positivity. Similarly, PI-RADS v2.1 scores (*p* < 0.001; OR = 9.779; 95% CI = 5.849-16.349) and PSAD (*p* < 0.001; OR = 6.128; 95% CI = 2.292-16.386) were independent risk factors for csPCa (**Table 3**).

**Table 3:** Univariate and multivariate logistic regression analysis.

Variables	Univariate analysis			Multivariate analysis			
	OR	95%CI	*P* value	B	OR	95%CI	*P* value
For PCa							
Age (years)	1.001	0.998-1.003	0.580	0.038	1.039	0.998-1.081	0.061
tPSA (ng/mL)	1.015	1.010-1.020	<0.001^*^	0.010	1.010	0.981-1.040	0.508
fPSA (ng/mL)	1.073	1.047-1.099	<0.001^*^	–0.973	0.933	0.808-1.077	0.344
fPSA/tPSA	0.409	0.162-1.033	0.059	–0.973	0.378	0.017-8.324	0.537
PV (cm^3^)	0.997	0.994-1.000	0.045^*^	–0.014	0.986	0.974-0.998	0.026^*^
PSAD (ng/(mL·cm³))	2.368	1.818-3.084	<0.001^*^	1.499	4.478	1.795-11.170	0.001^*^
PI-RADSv2.1 score	1.115	1.064-1.169	<0.001^*^	2.045	7.728	4.955-12.055	<0.001^*^
For csPCa							
Age (years)	1.031	1.007-1.056	0.011^*^	0.021	1.021	0.979-1.066	0.327
tPSA (ng/mL)	1.058	1.042-1.073	<0.001^*^	0.015	1.015	0.981-1.050	0.379
fPSA (ng/mL)	1.172	1.115-1.233	<0.001^*^	–0.098	0.906	0.776-1.059	0.216
fPSA/tPSA	0.021	0.002-0.176	<0.001^*^	–0.115	0.891	0.032-24.847	0.946
PV (cm^3^)	0.989	0.983-0.995	<0.001^*^	–0.014	0.986	0.972-1.000	0.056
PSAD (ng/(mL·cm³))	21.466	10.877-2.363	<0.001^*^	1.813	6.128	2.292-16.386	<0.001^*^
PI-RADS v2.1 score	13.945	8.941-21.751	<0.001^*^	2.280	9.779	5.849-16.349	<0.001^*^

csPCa, Clinically significant prostate cancer; fPSA, free prostate-specific antigen (PSA); tPSA, total PSA; f/tPSA, the ratio of free to total PSA; PI-RADS v2.1, Prostate Imaging Reporting and Data System version 2.1; PSAD, PSA density; PV, prostate volume; *P<0.05.

### ROC curve analysis for csPCa detection

**Figure 2:**
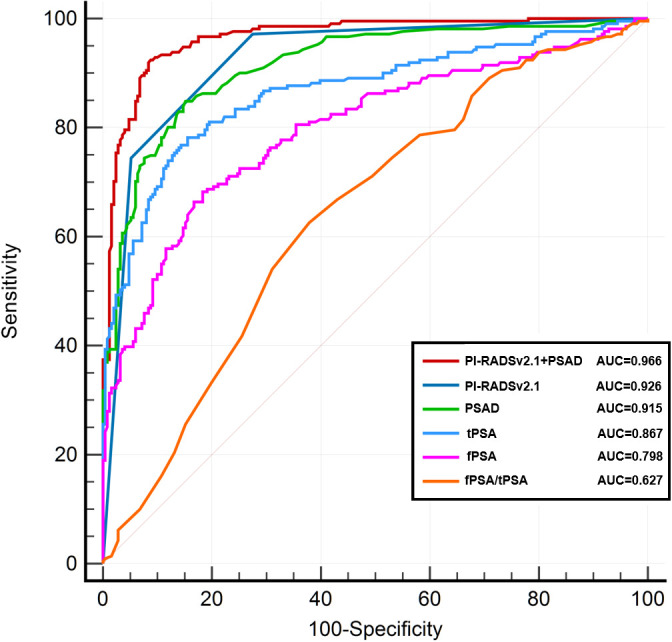
ROC curve analysis for csPCa detection.

**Figure 2** presents the ROC curves for detecting clinically significant prostate cancer (csPCa) using various indicators, including PI-RADS v2.1 score alone, PSAD, tPSA, and their combinations. Among them, the combination of PI-RADS v2.1 score and PSAD yielded the highest diagnostic performance, with an AUC of 0.966 (95% CI: 0.946–0.982), followed by PI-RADS v2.1 score alone (AUC = 0.926, 95% CI: 0.900–0.949), PSAD (AUC = 0.915, 95% CI: 0.888–0.938), and tPSA (AUC = 0.867, 95% CI: 0.832–0.898). Other indicators such as fPSA (AUC = 0.798), f/tPSA (AUC = 0.627), and prostate volume (PV, AUC = 0.673) demonstrated relatively lower discriminative ability. The combined model of PI-RADS v2.1 score + PSAD achieved a sensitivity of 92.4% and a specificity of 91.6%, outperforming the individual predictors.

To assess the statistical significance of AUC differences between models, we performed pairwise DeLong tests. The AUC of the combined model (PI-RADS v2.1 + PSAD) was significantly higher than that of PI-RADS v2.1 alone (p = 0.013) and PSAD alone (p = 0.025). Additionally, both PI-RADS v2.1 and PSAD showed significantly higher AUCs than tPSA (p < 0.001 for both comparisons), indicating their superior diagnostic value.

### Biopsy avoidance and detection rates by PI-RADS and PSAD thresholds

The diagnostic performance of PI-RADS v2.1 scoring as an indication for biopsy for PCa and csPCa is displayed in **Table 4**. When only patients with a score of ≥ 3 were biopsied, 13.9% (64/462) of patients could avoid biopsy, but 0.9% (2/233) of PCa cases were missed. The sensitivity for diagnosing csPCa was 99.1%, with an NPV of 96.9%. When only patients with a score of ≥ 4 were biopsied, 36.4% (168/462) of patients could avoid biopsy, but 6.0% (14/233) of PCa cases were missed, along with 2.8% (6/212) of csPCa cases. The sensitivity for diagnosing csPCa was 97.1%, with an NPV of 96.8%.

**Table 4:** Diagnostic performance of PI-RADS v2.1 score as an indication for biopsy for PCa and csPCa (cases, %).

PI-RADSv2.1	Sensitivity	Specificity	Jorden index	PPV	NPV	Avoid biopsy	Missed
For PCa							
≥ 3	99.1 (231/233)	27.1 (62/229)	0.26	58.0 (231/398)	96.9 (62/64)	13.9 (64/462)	0.9 (2/233)
≥ 4	94.0 (219/233)	75.1 (172/229)	0.69	79.9 (219/274)	91.7 (154/168)	36.4 (168/462)	6.0 (14/233)
5	69.1 (161/233)	96.1 (220/229)	0.65	89.4 (161/180)	57.8 (163/282)	61.0 (282/462)	26.6 (72/233)
For csPCa							
≥ 3	99.1 (209/211)	24.8 (62/251)	0.24	52.5 (209/398)	96.9 (62/64)	13.4 (62/462)	0.9 (2/211)
≥ 4	97.1 (205/211)	72.5 (182/251) 0.70	0.70	74.8 (205/274)	96.8 (182/188)	40.7 (188/462)	2.8 (6/211)
5	74.4 (157/211)	94.8 (238/251) 0.69	0.69	92.4 (157/170)	81.5 (238/292)	63.2 (292/462)	25.6 (54/211)

csPCa, Clinically significant prostate cancer; NPV, negative predictive value; PCa, prostate cancer; PI-RADS v2.1, Prostate Imaging Reporting and Data System version 2.1; PPV, positive predictive value.

### Comparison of tPSA and PSAD thresholds for csPCa detection

To evaluate the effectiveness of using serum markers alone in csPCa detection, we compared thresholds of tPSA and PSAD independently (**Table 5**).

**Table 5:** Comparison of diagnostic performance of various thresholds of tPSA and PSAD in detecting csPCa (cases, %).

Variable	Sensitivity	Specificity	Jorden index	PPV	NPV	Biopsies avoided	Cases missed
tPSA (ng/mL)							
≥ 4	100.0 (211/211)	1.6 (4/251)	0.16	46.2 (211/457)	100.0 (4/4)	0.9 (4/462)	0.0 (0/211)
≥ 10	92.4 (195/211)	41.8 (105/251)	0.34	57.2 (195/341)	86.8 (105/121)	26.2 (121/462)	7.6 (16/211)
PSAD [ng/ (mL·cm³)]							
≥ 0.15	98.1 (207/211)	34.7 (87/251)	0.33	55.8 (207/371)	95.6 (87/91)	19.7 (91/462)	1.8 (4/211)
≥ 0.30	90.5 (191/211)	72.1 (181/251)	0.63	73.2 (191/261)	90.0 (181/201)	43.5 (201/462)	9.5 (20/211)

csPCa, Clinically significant prostate cancer; NPV, negative predictive value; PPV, positive predictive value; PSAD, prostate-specific antigen (PSA) density; tPSA, total PSA.

At a threshold of tPSA ≥ 4 ng/mL, the sensitivity was comparable to PSAD ≥ 0.15 ng/(mL·cm³); however, PSAD markedly improved specificity (34.7% vs. 1.6%), allowing 87 fewer biopsies. Similarly, at higher thresholds [tPSA ≥ 10 ng/mL vs. PSAD ≥ 0.30 ng/(mL·cm³)], PSAD again provided higher specificity (72.1% vs. 41.8%) and reduced biopsies by 80 cases.

However, both PSAD thresholds led to four additional missed diagnoses of csPCa compared to tPSA, indicating that although PSAD improves specificity and reduces biopsy burden, its use alone risks missing significant disease. Notably, all four missed cases had PI-RADS v2.1 scores of 4–5, suggesting high suspicion on imaging. The integration of PSAD with PI-RADS v2.1 would have prevented these missed diagnoses, reinforcing the value of a combined diagnostic approach.

### Combined performance of PI-RADS v2.1 and PSAD in biopsy indications

**Table 6** illustrates the diagnostic performance of PI-RADS v2.1 scoring combined with PSAD as biopsy indications for csPCa. When the PI-RADS score was 1~2 and PSAD was < 0.30 ng/(mL·cm³), no cases of csPCa [0.0% (0/22)] were detected. When the PI-RADS score was 3 and PSAD was < 0.15 ng/(mL·cm³), no cases of csPCa [0.0% (0/48)] were detected. When the PI-RADS score was 4, the csPCa detection rate exceeded 10% (ranging from 11.8% to 100.0%) across all PSAD values in the table. When the score PI-RADS was 5 and PSAD was ≥0.30 ng/(mL·cm³), the csPCa detection rate was 95.5% (147/154). When the PI-RADS score was 5 and PSAD was ≥ 2.00 ng/(mL·cm³), the csPCa detection rate was 100% (64/64).

**Table 6:** Comparison of diagnostic performance of various thresholds of PI-RADS v2.1 score and PSAD in detecting csPCa (cases, %).

PSAD (ng/(mL·cm³))	PI-RADSv2.1 score			
	1~2	3	4	5
< 0.15	0 (0/22)	0 (0/48)	11.8 (2/17)	50.0 (2/4)
0.15~0.30	0 (0/52)	4.8 (2/42)	23.1 (6/26)	66.7 (8/12)
≥ 0.30	16.7 (2/12)	5.9 (2/34)	65.6 (40/61)	95.5 (147/154)
≥ 1.00	50.0 (1/2)	40.0 (2/5)	88.0 (22/25)	96.2 (102/106)
≥ 2.00	50.0 (1/2)	50.0 (1/2)	100.0 (12/12)	100.0 (64/64)

csPCa, Clinically significant prostate cancer; PI-RADS v2.1, Prostate Imaging Reporting and Data System version 2.1; PSAD, prostate-specific antigen density.

## Discussion

This study systematically validated PI-RADS v2.1 scores and PSAD as independent risk factors for clinically significant prostate cancer (csPCa) in an Asian population. Our findings confirmed that combining PI-RADS scoring with PSAD represents the optimal diagnostic strategy for biopsy indication, maintaining high sensitivity and specificity while potentially avoiding unnecessary biopsies in approximately 40% of patients. These results not only provide clear clinical guidelines for biopsy but also reduce unnecessary patient trauma and associated complications.

Our analysis revealed a significant stepwise increase in the detection rates of PCa and csPCa corresponding with increasing PI-RADS scores, consistent with findings by Oerther et al. (18) and Walker et al. (19), thereby affirming the general applicability and stability of the PI-RADS v2.1 scoring system across different populations. A meta-analysis by Park et al. (20) indicated high sensitivity (96%) and specificity (70%) for PI-RADS scores ≥ 4; our study demonstrated similar sensitivity but slightly higher specificity (72.5%). This discrepancy may be attributed to a lower proportion of benign prostatic hyperplasia (BPH) in our study population, highlighting potential population-specific differences in diagnostic performance.

In this study, PSAD demonstrated significantly higher diagnostic efficiency for csPCa compared with traditional total PSA (tPSA), with AUC values of 0.915 vs. 0.867, consistent with previous studies (21–26). High PSAD reflects increased tumor cell density and stromal activity per unit prostate volume, correlating with higher Gleason scores indicative of malignant lesions (21). Moreover, we observed notably larger prostate volumes in the benign group compared to PCa patients (58.50 cm³ vs. 38.25 cm³), suggesting that PSA dilution caused by BPH-related enlargement may reduce PSAD values. This finding aligns with Jia et al.’s report (21) on elevated PSA levels in patients with prostate hyperplasia. Some studies further suggest that BPH might compress tumor growth space, exerting a protective effect and negatively correlating with prostate cancer incidence (22). PSAD mitigates the confounding impact of BPH on PSA levels, thus directly reflecting the influence of csPCa on tPSA.

Although PSAD exhibited strong diagnostic performance, notably, 1.8% (4/211) of csPCa cases were still missed at a PSAD threshold of < 0.15 ng/(mL·cm³). Further analysis indicated that all four missed cases were patients with coexisting BPH and csPCa. Thus, while PSAD effectively excludes isolated BPH, it may inadvertently exclude certain csPCa cases with lower PSA and BPH severity. Fortunately, these four patients had PI-RADS scores of 4-5, and the combined application of PI-RADS scoring with PSAD successfully prevented missed diagnoses. This underscores the importance of not relying solely on PSAD for biopsy decisions, but combining it with PI-RADS scoring. ROC‐curve analysis further also showed that the combined model of PI-RADS v2.1 plus PSAD achieved the highest diagnostic accuracy (AUC = 0.966), Accordingly, we recommend the joint use of PI-RADS v2.1 and PSAD as a definitive indication for prostate biopsy.

Specifically, for patients with lower PI-RADS scores (1-3), PSAD significantly guides biopsy decisions. Biopsy recommendations for PI-RADS scores of 1–2 have varied; Ryoo et al. (27) suggested that these patients could safely avoid biopsy, whereas Anastay et al. (28) recommended biopsies or close monitoring for patients with PSAD ≥ 0.15 ng/(mL·cm³). Our findings indicated that patients with PI-RADS scores of 1–2 and PSAD < 0.30 ng/(mL·cm³) had zero csPCa cases (0/74), suggesting biopsy avoidance is safe. The potential reasons for these differences may be related to racial differences. A study (29) shows that the median tPSA of the East Asian race is higher than that of the Western race. However, csPCa detection rates significantly increased for PSAD ≥ 0.30 ng/(mL·cm³), highlighting PSAD’s role in identifying potential high-risk lesions. For PI-RADS score 3 patients, our results supported Görtz et al.’s recommendation (30), showing no csPCa cases below a PSAD threshold of < 0.15 ng/(mL·cm³), suggesting biopsy avoidance is safe; However, biopsies should be actively considered for patients with PSAD ≥ 0.15 ng/(mL·cm³) to minimize missed diagnoses.

For PI-RADS score 4 patients, our study revealed csPCa detection rates consistently exceeding 10% (range: 11.8%-100.0%), emphasizing the high-risk characteristics of a PI-RADS score of 4. These results align with previous research advocating biopsies for all such patients to avoid missing high-risk malignancies. Additionally, we found a 100% csPCa detection rate in patients with PI-RADS scores of 5 and PSAD ≥ 2.00 ng/(mL·cm³), suggesting that biopsy avoidance and direct non-invasive treatments such as hormone therapy, targeted therapy, immunotherapy, radiotherapy, or chemotherapy (31–33) could be explored with ethical approval. Similar recommendations by Emmett et al. (34) and Meissner et al. (35) propose direct radical treatment strategies without biopsy for patients highly suspicious for prostate cancer and PSMA PET/CT-positive lesions.

**Figure 3:**
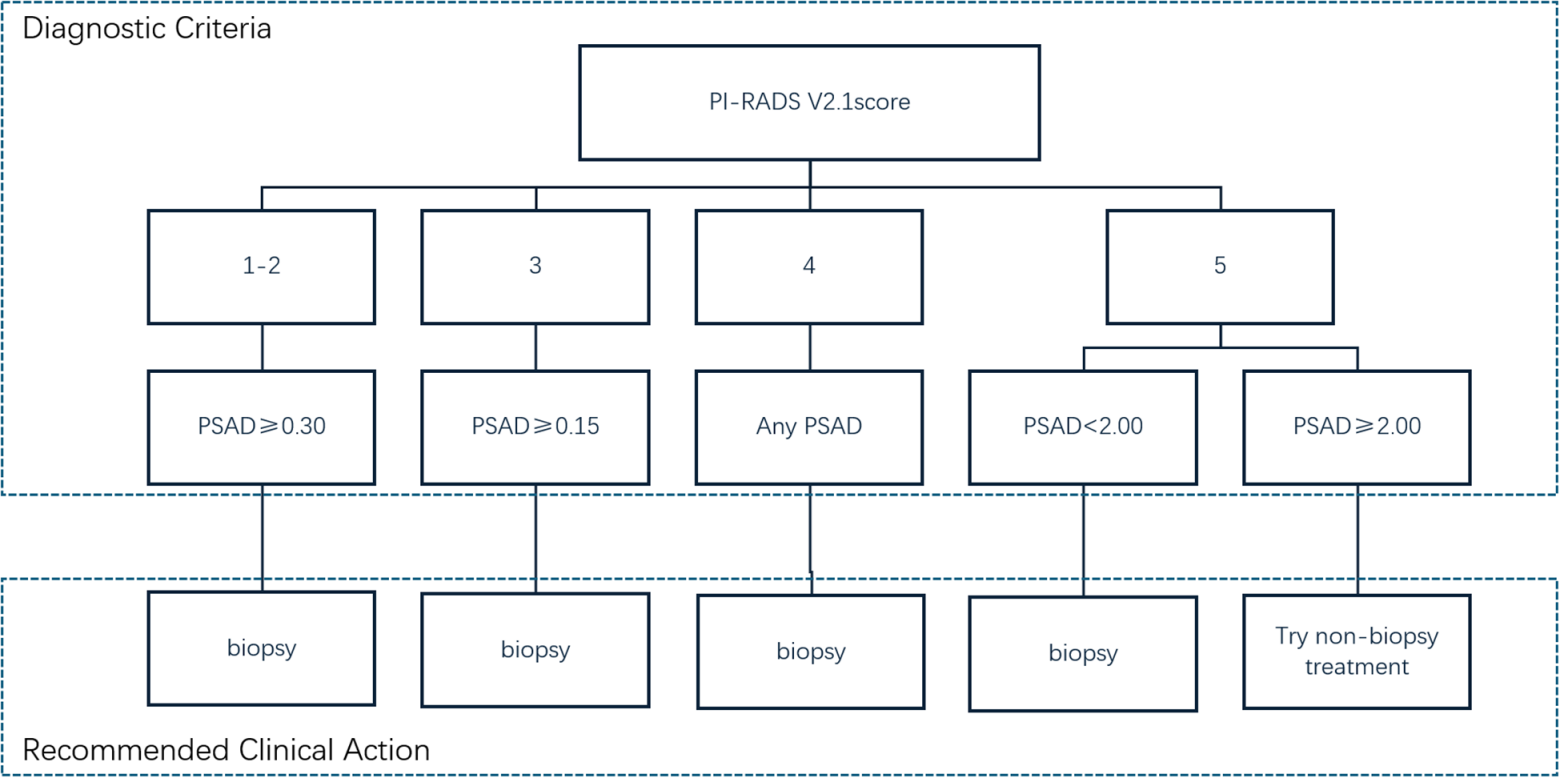
PI-RADS v2.1 combined with PSAD for guiding prostate biopsy indications.

Therefore, in our study, applying the combined strategy illustrated in **Figure 3** could have avoided unnecessary biopsies in 186 out of 462 patients (40.3%), including 74 patients with PI-RADS scores of 1–2, 48 with a score of 3, and 64 with a score of 5, thereby reducing biopsy-related discomfort and procedural risks for a substantial portion of the cohort.

This study had several limitations. First, biopsy is the gold standard for diagnosing csPCa but carries a risk of false negatives. Second, our cohort had relatively few patients with PI-RADS v2.1 scores of 1-3, possibly because clinicians considered the PI-RADS v2.1 score before deciding on a biopsy, thereby reducing the number of low-score biopsies. Finally, this was a retrospective study with potential patient selection bias. Future multicenter, prospective studies are needed to validate and generalize the results of this study.

## Conclusions

In conclusion, the proposed PI-RADS score combined with the PSAD grading strategy achieves a beneficial balance between preventing overtreatment and minimizing missed diagnoses. This approach can directly guide clinical decisions and be integrated into existing prostate cancer risk assessment tools (such as models recommended by EAU/AUA guidelines) to further optimize clinical pathways and biopsy decisions for MRI-negative or ambiguous cases. Additionally, PI-RADS scoring and PSAD could be incorporated into advanced artificial intelligence risk prediction models to facilitate individualized clinical decision-making.

## Data Availability

The original contributions presented in the study are included in the article/supplementary material. Further inquiries can be directed to the corresponding author.
